# Magnetohydrodynamic stagnation point on a Casson nanofluid flow over a radially stretching sheet

**DOI:** 10.3762/bjnano.11.114

**Published:** 2020-09-02

**Authors:** Ganji Narender, Kamatam Govardhan, Gobburu Sreedhar Sarma

**Affiliations:** 1 Department of Humanities and Sciences (Mathematics), CVR College of Engineering, Hyderabad, Telangana State, India; 2 Department of Mathematics, GITAM University, Hyderabad Campus, Telangana State, India

**Keywords:** Casson nanofluid, magnetohydrodynamics (MHD), stagnation point, thermal radiation, viscous dissipation

## Abstract

This article proposes a numerical model to investigate the impact of the radiation effects in the presence of heat generation/absorption and magnetic field on the magnetohydrodynamics (MHD) stagnation point flow over a radially stretching sheet using a Casson nanofluid. The nonlinear partial differential equations (PDEs) describing the proposed flow problem are reduced to a set of ordinary differential equations (ODEs) via suitable similarity transformations. The shooting technique and the Adams–Moulton method of fourth order are used to obtain the numerical results via the computational program language FORTRAN. Nanoparticles have unique thermal and electrical properties which can improve heat transfer in nanofluids. The effects of pertinent flow parameters on the nondimensional velocity, temperature and concentration profiles are presented. Overall, the results show that the heat transfer rate increases for higher values of the radiation parameter in a Casson nanofluid.

## Introduction

The heat transfer mechanism has been known for its significant importance in many fields of engineering and medical science in the last decades Since heat energy provides society with several benefits, the field of thermodynamics is applicable to and effectively connected with other fields. Heat transport processes plays a fundamental role in building design [1], fuel-filling systems [2], air compressor manufacturing [3], food industry [4], and in many other fields. In this regard, fluid dynamics is essential for regulating thermal energy through the usage of different fluids with good thermophysical properties. Research efforts have been focused on developing strategies to enhance thermal processes. For example, the fabrication of porous media, open and closed cavities and the implementation of magnetic effects, nanofluids and micrometer-sized channels have been employed to enhance thermal convection processes. Choi and collaborators [5] have used the term “nanofluid” for the first time to refer to a colloidal mixture of nanoparticles and a base fluid. Evidence has shown that metallic particles transfer more heat energy as compared to nonmetallic particles.

A Casson fluid is a non-Newtonian fluid in nature and therefore, behaves similarly to an elastic solid. When the stress rate is zero, the Casson fluid can be considered as a shear-thinning liquid, with infinite viscosity. On the other hand, when the stress rate approaches an infinite value the viscosity of the Casson fluid drops to zero [6]. Jam, tomato ketchup, honey, and concentrated fruit syrups are some quotidian examples of Casson fluids. In addition, Casson fluids have been implemented in the preparation of printing ink, silicon suspensions and polymers [7]. Over the past few years, a vast range of experiments and investigations have been carried out using Casson fluids due to their broad applicability in the scientific and engineering fields. Dash et al. [6] used a homogeneous porous medium inside a pipe to examine its flow behavior by using the Casson fluid model. The stagnation point flow for mixed convection and convective boundary conditions was analyzed by Hayat et al. also using the Casson fluid model [8]. In addition, Mukhopadhyay et al. [9] investigated the flow behavior over an unsteady stretching surface using the same approach. Moreover, different aspects of these flows were explored in other recent studies that applied the Casson fluid model to their systems [10–14].

The field of research in which the magnetic properties of electrically conducting fluids are studied is called magnetohydrodynamics (MHD). Magnetic fluids, liquids, metals and mixtures containing water, salt and other electrolytes are examples of materials that can be investigated via MHD. Hannes Alfen was the first to introduce the term MHD. MHD applies a sequence of Navier–Stokes equations and Maxwell’s equations to understand the flow behavior of a fluid with electromagnetic properties, as discussed by Chakraborty and Mazumdar [15]. Shah et al. [16] explored the MHD and heat transfer effects on the upper-convected Maxwell (UCM) fluid upon Joule heating and thermal radiation, using the Cattaneo–Christov heat flux model. Hayat et al. [17] investigated the mass exchange and MHD flow of a UCM fluid passing over an extended sheet. Ibrahim and Suneetha [18] studied the effects of Joule heating and viscous dissipation on steady Marangoni convective MHD flow over a surface in the presence of radiation.

The point in the flow field where the velocity of the fluid is zero is called the stagnation point. The study of viscous and incompressible fluids passing over a permeable plate or sheet is of great importance for the field of fluid dynamics. Over the past few decades, these studies have become even more important due to its applicability in manufacturing industries. The refrigeration of electronic instruments with a fan, cooling of atomic receptacles during an emergency power outage, and solar receivers for storage of thermal energy are a few examples in which viscous and incompressible fluids are directly applied. The study of a two-dimensional stagnation point flow was first investigated by Hiemenz [19]. Later on, Eckert [20] extended this problem by adding the energy equation in order to get a more accurate solution. In view of that, Mahapatra and Gupta [21], Ishak et al. [22], and Hayat et al. [23] have studied the effects of heat transfer at the stagnation point over a permeable plate. The MHD Casson fluid, including the effects of heat source/sink and convective boundary conditions was analyzed by Prabhakar et al. [24]. Besthapu et al. [25] examined the MHD stagnation flow of non-Newtonian fluids over a convective stretching surface. Ibrahim et al. [26] investigated the MHD stagnation point flow over a nonlinear stretching sheet by using a Casson nanofluid with velocity and convective boundary conditions. Ibrahim and Makinde [27] investigated the effect of slip and convective boundary conditions on a MHD stagnation point flow, considering heat transfer due to a Casson nanofluid passing over a stretching sheet.

Moreover, the flow analysis of nanofluids passing over radially stretched surfaces have many applications in several industry sectors, such as drawing of plastic films, manufacturing of glass, production of paper, and refining crude oil. Recently, many researchers have been focusing their attention on nanoparticles, since they exhibit remarkable electrical, optical, and chemical properties in addition to having Brownian motion and thermophoretic properties. Due to these features, nanoparticles are widely used in catalysis, imaging, energy-based research, microelectronics, and in other applications in the medical and environmental fields. These nanoparticles are composed of metals and nonmetals and are frequently infused into heat transfer fluids (e.g., water, diethylene glycol and propylene glycol) to increase their efficiency. Rafique et al. [28] studied the impact of Casson nanofluid boundary layer flow over an inclined extending surface, considering Soret and Dufour effects. In addition, Rafique et al. [29] studied the impact of Brownian motion and thermophoresis diffusion on Casson nanofluid boundary layer flow over a nonlinear inclined stretching sheet. An unsteady flow of a Casson fluid along a nonlinear stretching surface was studied by Ullah et al. [30]. A Casson fluid over a non-isothermal cylinder, subjected to suction/blowing was analyzed by Ullah et al. [31]. Moreover, various researchers have been investigating the Casson fluid model for different flow problems [32–36].

Motivated by the previous findings on non-Newtonian and Newtonian fluids, the study of the stagnation point MHD flow using Casson nanofluids has been presented. The governing partial differential equations (PDEs) have been converted to a set of ordinary differential equations (ODEs) through suitable similarity transformations and the numerical solution has been derived by the shooting method.

## Mathematical Modelling

**Figure 1:**
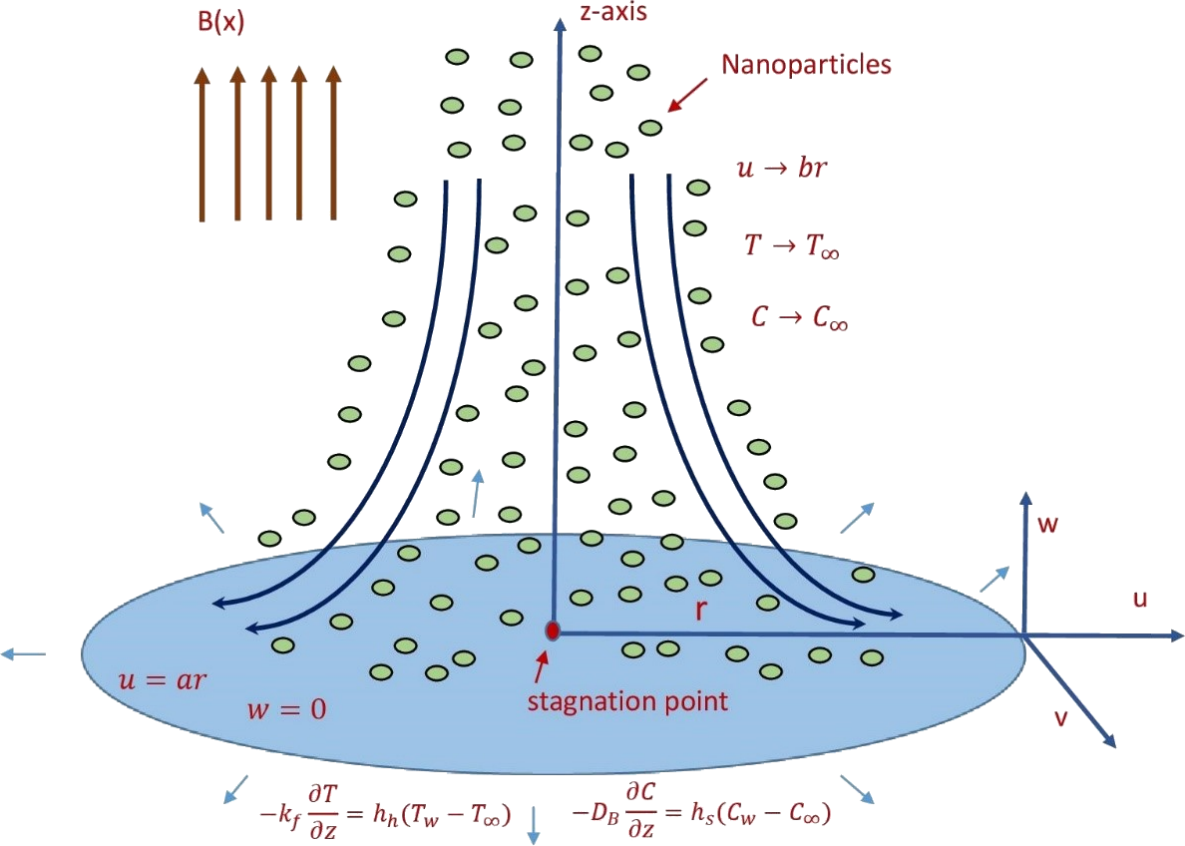
Schematic of the physical model in which a Casson nanofluid is passing a radially stretched surface in the proximity of a stagnation point.

The present model aims to investigate the laminar, incompressible and steady flow of a Casson nanofluid passing a radially stretched surface in the proximity of a stagnation point. Considering the thermal radiation and heat generation/absorption effects, the characteristics of the flow and heat transfer are examined. The coordinate system is chosen such that the *r*-axis is along the direction of the flow whereas the *z*-axis is perpendicular to the flow direction (Figure 1).

The velocity of the outer flow is designated as *U*_e_ and the direction of the uniform magnetic field is chosen to be normal to the surface of the fluid flow. The Brownian motion and thermophoretic effects have been considered as well as the convective surface conditions. A convective heating process is applied to regulate the sheet temperature *T_w_*. The nanoparticle concentration, *C_w_*, is assumed to be constant. When *y* values tend to infinity, the concentration and temperature of the nanofluid is represented by *C*_∞_ and *T*_∞_, respectively. The constitutive equations of the Casson nanofluid model are described as follows [10–14].

Firstly, the continuity equation considers the physical principle of mass conservation:

[1] $$\frac{\partial u}{\partial r}+\frac{u}{r}+\frac{\partial w}{\partial z}=0.$$

In addition, the momentum is described by Newton’s second law:

[2] $$\frac{\partial u}{\partial r}u+\frac{\partial v}{\partial z}w=-\frac{\sigma B_{0}}{\rho_{f}}\left(u-U_{e}\right)+\nu_{f}\left(1+\frac{1}{\beta }\right)\left(\frac{\partial^{2}u}{\partial r^{2}}+\frac{\partial^{2}u}{\partial z^{2}}\right).$$

Following the principles of conservation of energy, the energy equation is written as

[3] $$\begin{array}{l} \frac{\partial T}{\partial r}u+\frac{\partial T}{\partial z}w=-\frac{\nu_{f}}{\rho_{p}}\left(1+\frac{1}{\beta }\right){\left(\frac{\partial u}{\partial z}\right)}^{2}+\alpha_{f}\left(\frac{\partial^{2}T}{\partial r^{2}}+\frac{\partial^{2}T}{\partial z^{2}}\right)\\ \;\;\;\;\;\;\;\;-{\frac{1}{\left(\rho c_{p}\right)}}_{f}\frac{\partial q_{r}}{\partial z}\\ \;\;\;\;\;\;\;\;+\tau \left\{D_{B}\left(\frac{\partial C}{\partial z}\frac{\partial T}{\partial z}+\frac{\partial C}{\partial r}\frac{\partial T}{\partial r}\right)+\frac{D_{T}}{T_{\infty }}{\left(\frac{\partial T}{\partial r}\right)}^{2}+{\left(\frac{\partial T}{\partial z}\right)}^{2}\right\}\\ \;\;\;\;\;\;\;\;+\frac{Q_{0}}{{\left(\rho c_{p}\right)}_{f}}\left(T-T_{\infty }\right)+\frac{\sigma B_{0}^{2}{\left(u-U_{e}\right)}^{2}}{{\left(\rho c_{p}\right)}_{f}}, \end{array}$$

and the mass transfer equation is

[4] $$\begin{array}{c} \frac{\partial C}{\partial r}u+\frac{\partial C}{\partial z}w=D_{B}\left(\frac{\partial^{2}C}{\partial r^{2}}+\frac{\partial^{2}C}{\partial z^{2}}\right)-\frac{D_{T}}{T_{\infty }}\left(\frac{\partial^{2}T}{\partial r^{2}}+\frac{\partial^{2}T}{\partial z^{2}}\right)\\ -C_{r}^{}\left(C-C_{\infty }\right). \end{array}$$

The corresponding boundary conditions at the boundary surface are

[5] $$\begin{array}{l} u=U_{w}\left(x\right)=ar,w=0,-k_{f}\frac{\partial T}{\partial z}=h_{h}\left(T_{f}-T\right),\\ -D_{\text{B}}\frac{\partial C}{\partial z}=h_{s}\left(C_{f}-C\right)\text{ at }z=0 \end{array}$$

[6] $$u\to U_{e}=br,T\to T_{\infty },C\to C_{\infty }\text{ as }z\to \infty$$

In the previous equations, ν*_f_* is the kinematic viscosity, ρ*_f_* is the fluid density, α represents the thermal diffusivity, *C_p_* represents a constant pressure for a specific heat value, *k*_0_ denotes a chemical reaction coefficient, (ρ*c_p_*)*_f_* represents the heat capacity, *D*_B_ represents the Brownian diffusion coefficient, *Q*_0_ represents the volumetric heat generation, *D_T_* is the thermophoresis diffusion coefficient, σ is the electrical conductivity, β represents the Casson fluid parameter, and *T* represents the nanofluid temperature.

The following similarity variables are taken into consideration:

[7] $$\begin{array}{l} \eta =z\sqrt{\frac{a}{\nu_{f}},}\;\;u=arf^{'}\left(\eta \right),\;w=-2\sqrt{a\nu_{f}}f\left(\eta \right),\\ \theta \left(\eta \right)=\frac{T-T_{\infty }}{T_{f}-T_{\infty }},\;\;\phi \left(\eta \right)=\frac{C-C_{\infty }}{C_{w}-C_{\infty }}. \end{array}$$

Finally, the ODEs describing the proposed flow problem can be written as

[8] $$\left(1+\frac{1}{\beta }\right)f^{'''}+2ff^{"}-{\left(f^{'}\right)}^{2}+A^{2}-Ha\left(f^{'}-A\right)=0,$$

[9] $$\left(1+\frac{4}{3}R\right)\theta^{"}+\mathrm{Pr}\left(\begin{array}{l} f\theta^{'}+Nb\phi^{'}\theta^{'}+Nt{\left(\theta^{1}\right)}^{2}\\ +\left(1+\frac{1}{\beta }\right)Ec{\left(f^{"}\right)}^{2}\\ +Q\theta +EcM^{2}{\left(f^{'}-A\right)}^{2} \end{array}\right)=0,$$

[10] $$\phi^{"}+Sc\left(2f\phi^{'}-\gamma \phi \right)+\frac{Nt}{Nb}\theta^{"}=0.$$

The transformed boundary conditions are

[11] $$\begin{array}{l} f(0)=0,f^{'}\left(0\right)=\lambda ,\theta^{'}\left(0\right)=-Bi1\left[1-\theta \left(0\right)\right],\\ \phi^{'}\left(0\right)=-Bi2\left[1-\phi \left(0\right)\right]\text{ at }\;y=0\\ f^{'}\left(\infty \right)\to A,\theta \left(\infty \right)\to 0,\phi \left(\infty \right)\to 0\;\text{ as }\;\eta \to \infty \end{array}\}$$

and the dimensionless parameters are defined as

[12] $$\begin{array}{l} Nb=\frac{\tau D_{B}\left(C_{f}-C_{\infty }\right)}{\nu_{f}},\mathrm{Pr}=\frac{\nu_{f}}{\alpha },Nt=\frac{\tau D_{T}\left(T_{f}-T_{\infty }\right)}{\nu_{f}T_{\infty }},\\ Ha=\frac{\sigma B_{0}^{2}}{\rho a},Q=\frac{Q_{0}}{\rho c_{p}a},R=\frac{4T_{\infty }\sigma^{*}}{k^{*}\left(\alpha \rho c_{p}\right)},\\ Ec=\frac{a^{2}r^{2}}{\alpha c_{p}\left(T_{f}-T_{\infty }\right)},A=\frac{b}{a},Sc=\frac{\nu_{f}}{D_{B}},\\ \gamma =\frac{C_{r}}{a},Bi1=\frac{h_{h}}{k_{f}}\sqrt{\frac{\nu_{f}}{a}},Bi2=\frac{h_{s}}{D_{B}}\sqrt{\frac{\nu_{f}}{a}}. \end{array}\}$$

The formulas for the dimensional form of the skin-friction coefficient *C_f_*, the Nusselt number *Nu*, and Sherwood number *Sh*, are given by

[13] $$C_{f}=\frac{\tau_{w}}{\rho_{f}U_{w}^{2}},Nu=\frac{rq_{w}}{k_{f}\left(T_{f}-T_{\infty }\right)},Sh=\frac{rq_{w}}{D_{B}\left(\phi_{f}-\phi_{\infty }\right)}$$

and the formulas for τ*_w_*, *q_w_*, and *q_m_* are

[14] $$\begin{array}{l} \tau_{w}=\mu \left(1+\frac{1}{\beta }\right){\left(\frac{\partial u}{\partial z}\right)}_{z=0},\;q_{w}=-k_{f}{\left(\frac{\partial T}{\partial z}-\frac{q_{r}}{k_{f}}\right)}_{z=0},\\ q_{m}=-D_{B}{\left(\frac{\partial C}{\partial z}\right)}_{z=0} \end{array}$$

The result of the transformation of the above formulas into their dimensionless form is

[15] CfRe=(1+1β)f"(0),NuRe=−[1+43R]−θ'(0),ShRe−ϕ'(0)

where Re = *rU_w_*/ν*_f_* is the local Reynolds number and ν*_f_* = µ/ρ is the kinematic viscosity.

## Solution Methodology

In order to solve the system of ODEs (Equation 8–Equation 10) subjected to the boundary conditions (Equation 11) the shooting technique has been used. First, Equation 8 is solved numerically and then the computed results of *f*, *f*' and *f*''are used in Equation 9 and Equation 10. For the numerical treatment of Equation 8, the missing initial condition, *f*''(0), has been denoted as *s* and the following notations have been considered:

[16] $$f=h_{1},f^{'}=h_{2},f^{"}=h_{3},\frac{\partial f}{\partial s}=h_{4},\frac{\partial f^{'}}{\partial s}=h_{5},\frac{\partial f^{"}}{\partial s}=h_{6}.$$

Using the previous notations, Equation 8 can be converted into a system of three first-order ODEs. The first three of the following ODEs correspond to Equation 8 and the other three are obtained by differentiating the first three equations with respect to *S*:


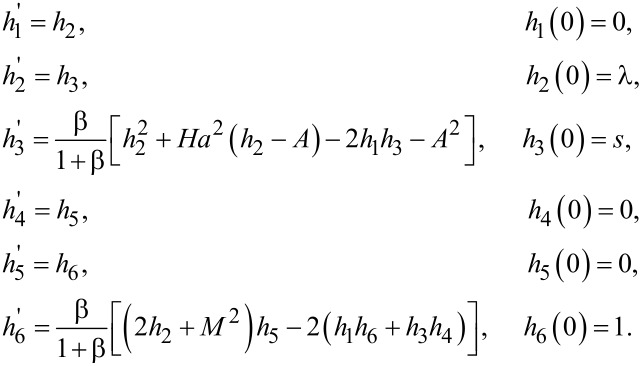


The Adams–Bashforth–Moulton method has been used to solve the previous initial value problem. In order to get the approximate numerical results, the domain of the problem was bounded (i.e., [0, η_∞_], where η_∞_ is chosen to be an appropriate finite positive real number such that the variation in the solution for η > η_∞_ can be ignored). The missing condition for the previous system of equations is chosen such that (*h*_2_(η_∞_))_s_ = *A*. This algebraic equation is solved by using the Newton’s method governed by the following iterative formula:

[17] $$\begin{array}{l} s^{\left(n+1\right)}=s^{\left(n\right)}-\frac{{\left(h_{2}\left(\eta_{\infty }\right)\right)}_{s=s^{\left(n\right)}}-A}{{\left(\frac{\partial h_{2}\left(\eta_{\infty }\right)}{\partial s}\right)}_{s=s^{\left(n\right)}}}\\ \Rightarrow s^{\left(n+1\right)}=s^{\left(n\right)}-\frac{{\left(h_{2}\left(\eta_{\infty }\right)\right)}_{s=s^{\left(n\right)}}-A}{{\left(h_{5}\left(\eta_{\infty }\right)\right)}_{s=s^{\left(n\right)}}}. \end{array}$$

The stopping condition for the shooting method is set as

[18] $$\left|{\left(h_{2}\left(\eta_{\infty }\right)\right)}_{s=s^{\left(n\right)}}-A\right|<\varepsilon ,$$

in which ε is a very small positive number.

Now to solve Equation 9 and Equation 10 numerically, the missing initial conditions, θ(0) and ϕ(0), have been denoted by *l* and *m*, respectively. Therefore, after taking the new notation into account, we have

[19] $$\begin{array}{l} \theta =y_{1},\theta^{'}=y_{2},\phi^{'}=y_{3},\phi^{"}=y_{4},\frac{\partial \theta }{\partial l}=y_{5},\frac{\partial \theta^{'}}{\partial l}=y_{6},\frac{\partial \phi }{\partial l}=y_{7}\\ \frac{\partial \phi^{'}}{\partial l}=y_{8},\frac{\partial \theta }{\partial m}=y_{9},\frac{\partial \theta^{'}}{\partial m}=y_{10},\frac{\partial \phi }{\partial m}=y_{11},\frac{\partial \phi^{'}}{\partial m}=y_{12}. \end{array}\}.$$

By incorporating the above notations, a system of first order ODEs is achieved, as follows:


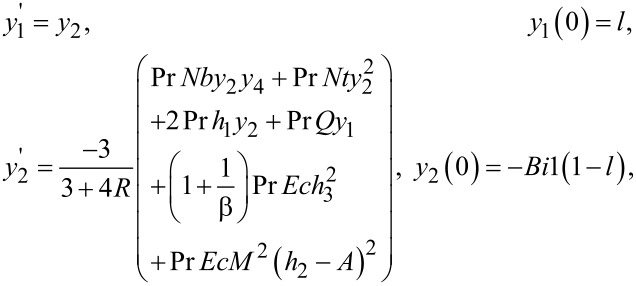



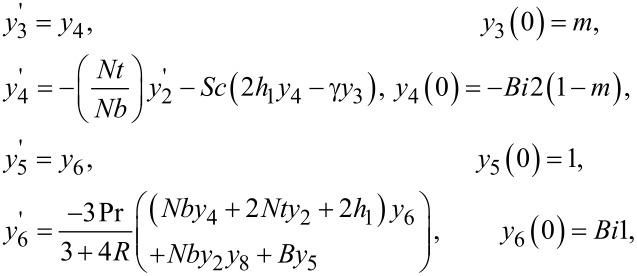



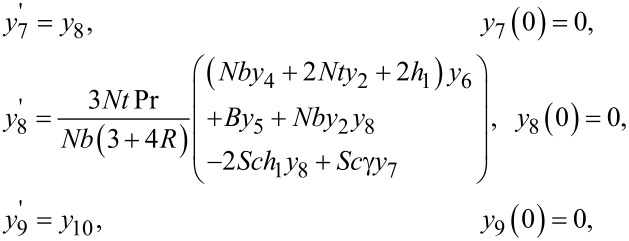



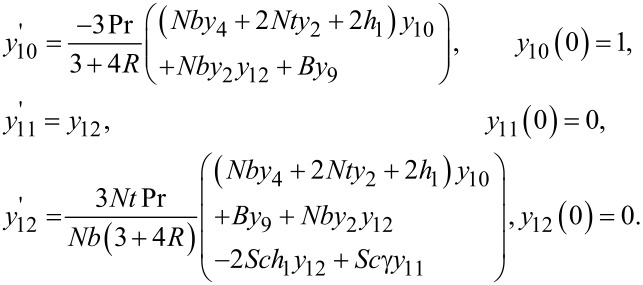


The Adams–Bashforth–Moulton method has been taken into account to solve the initial value problem. For the previous system of equations, the missing conditions were chosen, such that

[20] $${\left(y_{1}\left(l,m\right)\right)}_{\eta =\eta_{\infty }}=0,{\left(y_{3}\left(l,m\right)\right)}_{\eta =\eta_{\infty }}=0.$$

The above algebraic equations have been solved by using Newton’s method governed by the following iterative formula:


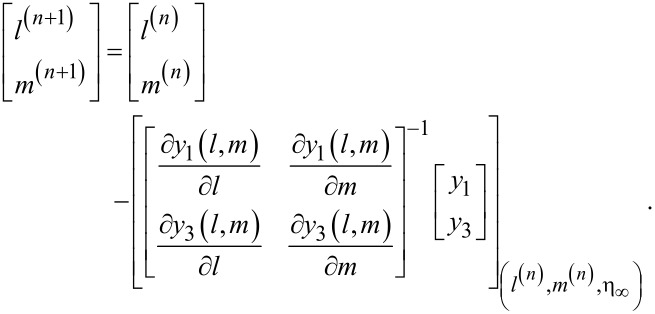


The stopping criteria for the shooting method is set as:

[21] $$\max\left\{\left|y_{1}\left(\eta_{\infty }\right)\right|,\left|y_{3}\left(\eta_{\infty }\right)\right|\right\}<\varepsilon ,$$

in which ε is set as a very small positive number. In this work, ε is set as 10^−5^ whereas η_∞_ is set as 7.

## Results and Discussion

In this section, the numerical results of the skin-friction coefficient, Nusselt and Sherwood numbers are listed in tables and shown in graphs. The different values obtained depend on the flow parameters chosen. The physical parameters have the following admissible ranges: 0 ≤ *Ha* ≤ 2, 0.3 ≤ *A* ≤ 2.5, 0.1 ≤ β ≤ 1.5, 0.3 ≤ Pr ≤ 2.0, 0.5 ≤ *Ec* 2.5, 0.1 ≤ *R* ≤ 0.5, 0.1 ≤ *Q* ≤ 0.5, 0.3 ≤ *Sc* ≤ 0.6, 0.1 ≤ γ ≤ 2.0, 0.1 ≤ *Nt* ≤ 2.0, 0.1 ≤ *Nb* ≤ 2.0, γ = 1.0, 1 ≤ *Bi*1 ≤ 2.0, and 1 ≤ *Bi*2 ≤ 2.0.

### Skin-friction coefficient, Nusselt and Sherwood numbers

Prabhakar et al. [24] used a fourth-order Runge–Kutta method to obtain the numerical solution of the discussed model, whereas Attia [37] used the shooting technique and the computational software MATLAB. In the present study, the shooting technique along with the fourth order Adams–Moulton method were used to reproduce the previously published solution [24,37].

To validate the code written in the computational program language Fortran, the results of –*f*''(0) and –θ'(0) were reproduced for the problem discussed by Attia [37] and Prabhakar et al. [24].

Tables 1–3 show that there is an excellent agreement between the results yielded by the present code and the previously published results.

**Table 1:** Comparison between the computed values of *f*''(0) and the values given by Attia [37], when *Nt* = *Nb* = *R* = *Ec* = *Sc* = 0.

*Ha*	*A*	*f*''(0)		*Ha*	*A*	*f*''(0)	
	
		Attia	Present			Attia	Present

0	0.1	−1.1246	−1.1246260	2	0.1	−2.1138	−2.1137140
	0.2	−1.0556	−1.0555810		0.2	−1.9080	−1.9079860
	0.5	−0.7534	−0.7534078		0.5	−1.2456	−1.2455380
	1.0	0.0000	0.0000000		1.0	0.0000	0.0000000
	1.1	0.1821	0.1820637		1.1	0.2691	0.2690781
	1.2	0.3735	0.3735214		1.2	0.5445	0.5445290
	1.5	1.0009	1.0008780		1.5	1.4080	01.4080270
1	0.1	−1.4334	−1.4334070	3	0.1	−2.9174	−2.9173560
	0.2	−1.3179	−1.3178900		0.2	−2.6141	−2.6140730
	0.5	−0.9002	−0.9001369		0.5	−1.6724	−1.6723740
	1.0	0.0000	0.0000000		1.0	0.0000	0.0000000
	1.1	0.2070	0.2070196		1.1	0.3494	0.3494373
	1.2	0.4004	0.4223360		1.2	0.7037	0.7037439
	1.5	1.1157	1.1156770		1.5	1.7954	1.7954280

**Table 2:** Comparison between the computed results of Nusselt number –θ'(0) and the results given by Attia [37], when *Nt* = *Nb* = *R* = *Ec* = *Sc* = 0.

Pr	*A*	–θ'(0)		Pr	*A*	–θ'(0)	
	
		Attia	Present			Attia	Present

0.05	0.1	0.1273	0.166529400	0.5	0.1	0.4691	0.476318600
	0.2	0.1421	0.175023100		0.2	0.5223	0.526475900
	0.5	0.1845	0.201851100		0.5	0.6345	0.633877500
	1.0	0.2439	0.247389100		1.0	0.7699	0.764000400
	1.1	0.2545	0.256288100		1.1	0.7933	0.786525000
	1.2	0.2632	0.265061900		1.2	0.8136	0.808239000
	1.5	0.2919	0.290530900		1.5	0.8793	0.849610600
0.1	0.1	0.1618	0.194615100	1	0.1	0.7657	0.772774200
	0.2	0.1911	0.212448800		0.2	0.8152	0.818562500
	0.5	0.2615	0.265139300		0.5	0.9332	0.929409300
	1.0	0.3343	0.342184300		1.0	1.0888	1.077056000
	1.1	0.3581	0.355768200		1.1	1.1166	1.103455000
	1.2	0.3700	0.368815700		1.2	1.1408	1.129085000
	1.5	0.4080	0.405144700		1.5	1.2200	1.202041000

**Table 3:** Comparison between the computed values of *f*''(0) and the results given by Prabhakar et al. [24].

λ	*Ha*	*f*''(0)	

		Prabhakar et al. [24]	Present

0	1.0	1.64532	1.645239000
0.2	1.0	1.38321	1.383139000
0.5	1.0	0.92353	0.923487700
0.5	0.0	0.78032	0.780284500
	1.0	0.92353	0.923487700
	5.0	1.35767	1.357532100
	10.0	1.75768	1.757437000

Table 4 and Table 5 show the numerical results of the skin-friction coefficient along with the Nusselt and Sherwood numbers for the present model, taking into account changes in the values of various parameters, such as β, *Ha*, *R*, *A*, Pr, *Q*, *Nb*, *Nt*, *Ec* and *Sc*.

**Table 4:** The computed results for the skin-friction coefficient, Nusselt and Sherwood numbers, for γ = 1, *Bi*1 = 0.1 = *Bi*2, where *a*_1_ = (1 + 1/β) and *a*_2_ = (1 + 4/3·*R*).

β	*Ha*	*A*	*R*	Pr	*Q*	*Nb*	*Nt*	*Ec*	*Sc*	−*a*_1_*f*´´(0)	−*a*_2_θ´(0)	−ϕ´(0)

0.5	1.0	0.1	0.1	0.7	0.1	0.5	0.1	0.1	1.2	2.485303	0.0859357	0.0939868
5.0	–	–	–	–	–	–	–	–	–	1.570312	0.0860365	0.0937332
10	–	–	–	–	–	–	–	–	–	1.503451	0.0859404	0.0937079
–	1.2	–	–	–	–	–	–	–	–	2.688387	0.083406	0.0940580
–	1.4	–	–	–	–	–	–	–	–	2.911371	0.0805399	0.0941407
–	–	0.3	–	–	–	–	–	–	–	2.0619300	0.0913473	0.0938944
–	–	0.5	–	–	–	–	–	–	–	1.5593120	0.0942490	0.0938664
–	–	–	0.2	–	–	–	–	–	–	2.4853030	0.0954124	0.0939689
–	–	–	0.3	–	–	–	–	–	–	2.4853030	0.1047357	0.0939546
–	–	–	–	1.0	–	–	–	–	–	2.4853030	0.0871634	0.0940612
–	–	–	–	2.0	–	–	–	–	–	2.4853030	0.0874003	0.0942927
–	–	–	–	–	0.5	–	–	–	–	2.4853030	0.0688995	0.0944750
–	–	–	–	–	0.7	–	–	–	–	2.4853030	0.1130105	0.0935984
–	–	–	–	–	–	0.7	–	–	–	2.4853030	0.0858337	0.0939400
–	–	–	–	–	–	0.8	–	–	–	2.4853030	0.0857826	0.0939254
–	–	–	–	–	–	–	0.2	–	–	2.4853030	0.0858089	0.0941674
–	–	–	–	–	–	–	0.3	–	–	2.4853030	0.0856807	0.0943550
–	–	–	–	–	–	–	–	0.5	–	2.4853030	0.0366119	0.0959349
–	–	–	–	–	–	–	–	1.0	–	2.4853030	−0.025837	0.0983850
–	–	–	–	–	–	–	–	–	1.4	2.4853030	0.0859497	0.0944484
–	–	–	–	–	–	–	–	–	1.6	2.4853030	0.0859616	0.0948187

**Table 5:** The computed results of the skin-friction coefficient, Nusselt and Sherwood numbers for β = 0.5, *Ha* = 1, *A* = 0.1, *R* = 0.1, Pr = 0.7, *Q* = 0.1, *Nt* = 0.1, *Nb* = 0.5, *Ec* = 0.1, *Sc* = 1.2, where *a*_1_ = (1 + 1/β) and *a*_2_ = (1 + 4/3·*R*).

**γ**	*Bi*1	*Bi*2	−*a*_1_*f*''(0)	−*a*_2_θ'(0)	−ϕ'(0)

1.0	0.1	0.1	2.485303	0.0859357	0.0939868
1.5	–	–	2.485303	0.0859589	0.0946673
2.0	–	–	2.485303	0.0859759	0.0951565
–	0.2	–	2.485303	0.1514275	0.0937815
–	0.3	–	2.485303	0.2029085	0.0936212
–	–	0.2	2.485303	0.0857094	0.1770382
–	–	0.3	2.485303	0.0855062	0.2509579

### Velocity, temperature and concentration

**Figure 2:**
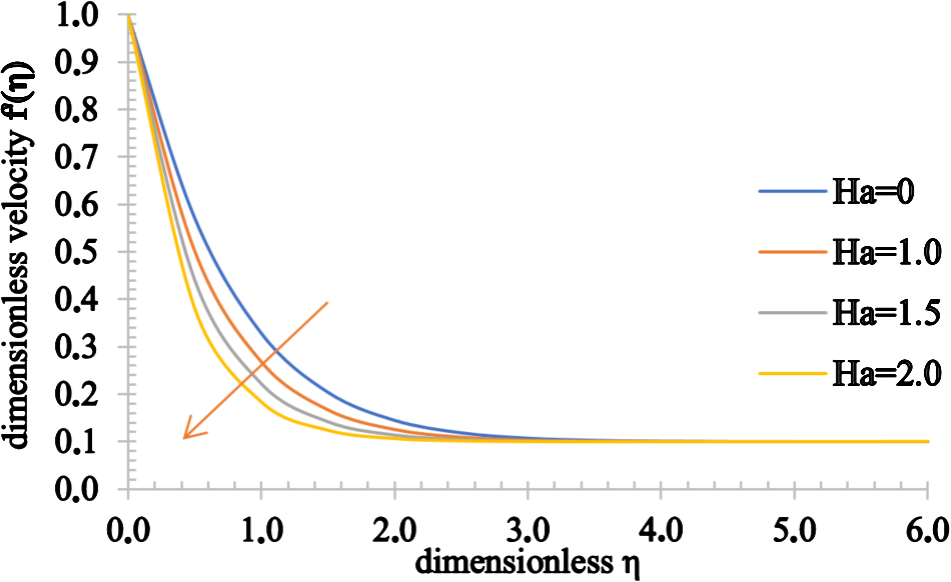
The velocity profile of a fluid for increasing values of the Hartmann number, *Ha*.

**Figure 3:**
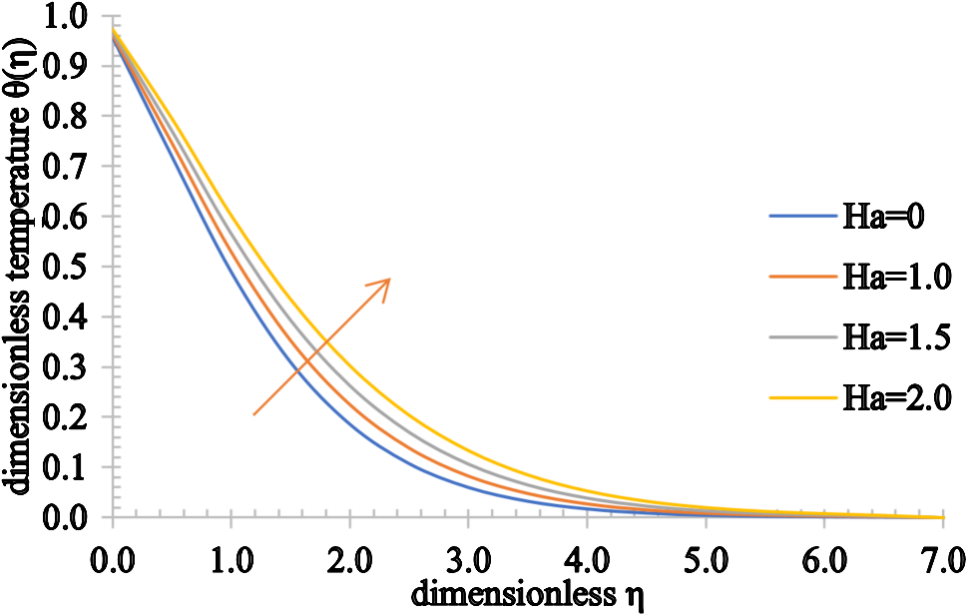
The temperature profile of a fluid for increasing values of the Hartmann number, *Ha*.

**Figure 4:**
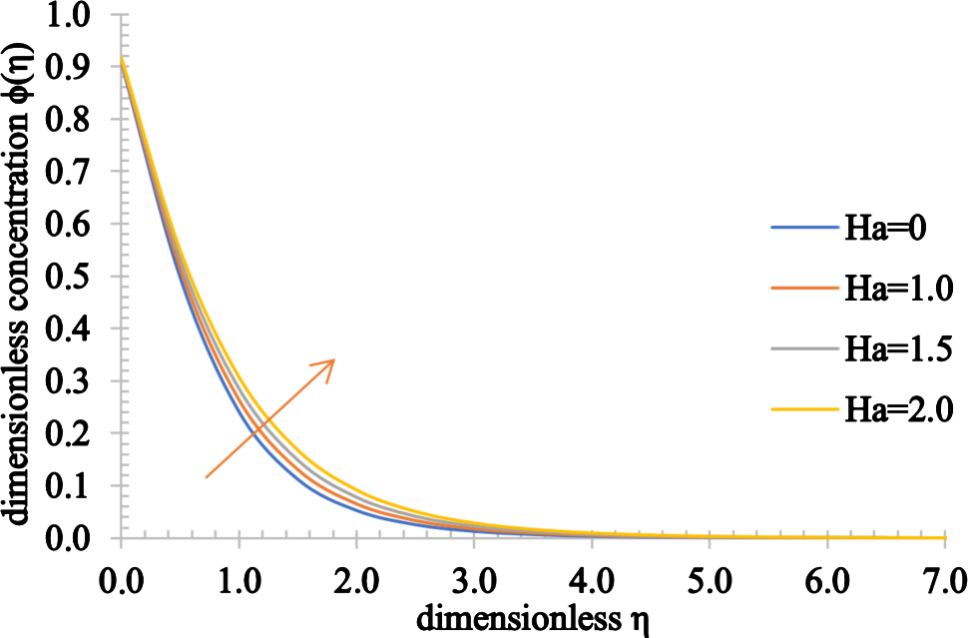
The concentration profile of a fluid for increasing values of the Hartmann number, *Ha*.

Figures 2–4 present the influence of the Hartmann number on the velocity, temperature and concentration distributions. For high values for *Ha*, the fluid velocity decreases while the temperature and concentration of the fluid increase. This stems from the fact that an opposing force generated by the magnetic field, generally referred to as the Lorentz force, reduces the fluid motion, resulting in a reduction in the momentum boundary layer thickness and an increase in the thermal and concentration boundary layer thickness values.

**Figure 5:**
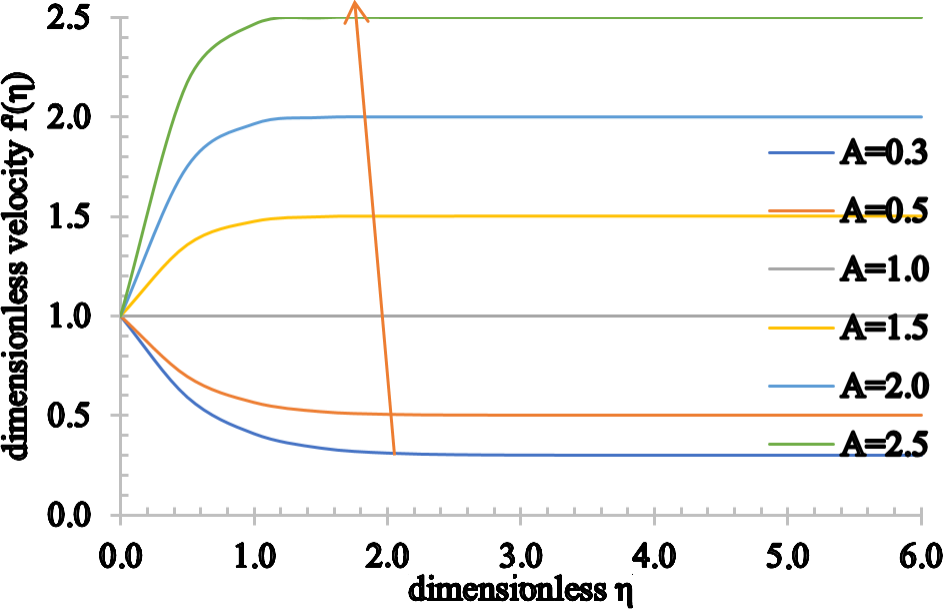
The velocity profile for increasing values of *A*.

**Figure 6:**
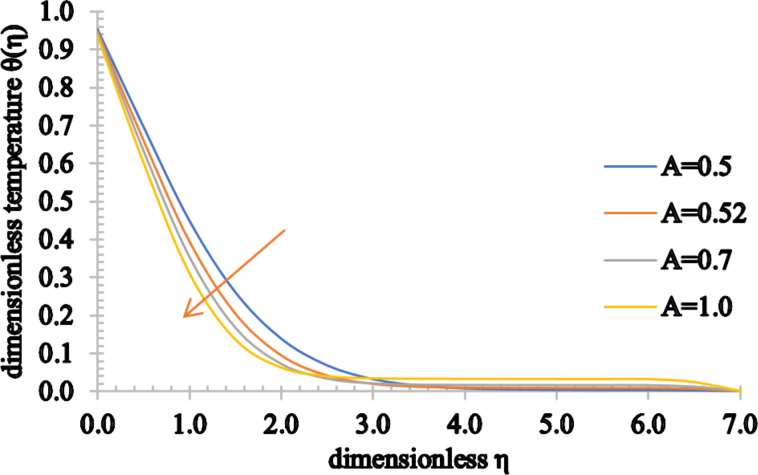
The temperature profile for increasing values of *A*.

**Figure 7:**
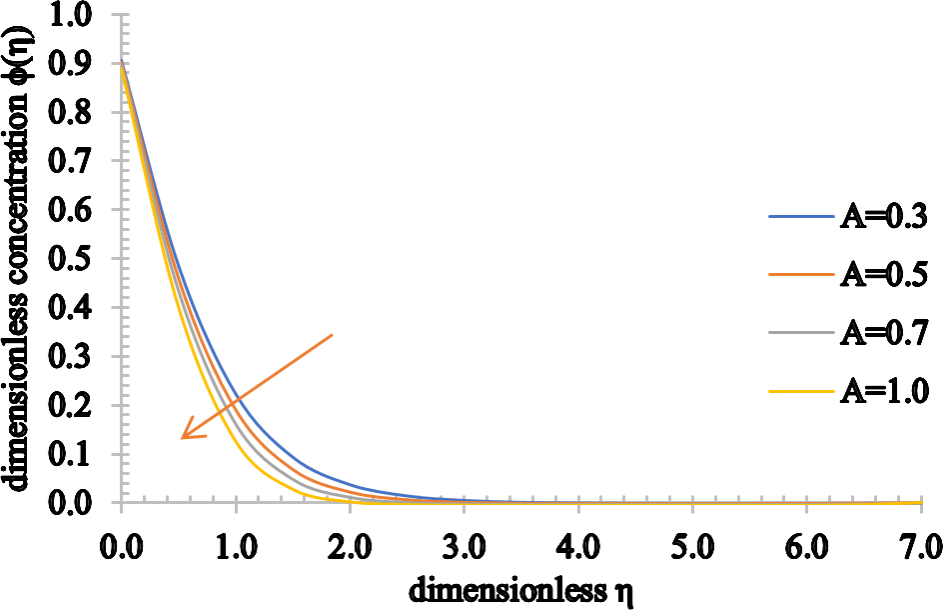
The concentration profile for increasing values of *A*.

Figures 5–7 show the effect of *A* on the velocity, temperature and concentration distributions. An increase in the flow velocity is observed for *A* > 1, whereas a reduction in the flow velocity is observed for *A* < 1. Also, both the temperature and concentration profiles decrease when *A* assumes higher values. As the value of *A* increases, the heat transfer from the sheet to the fluid reduces and, as a result, the temperature significantly decreases. Furthermore, the thermal boundary layer thickness is reduced as well as the concentration boundary layer thickness.

**Figure 8:**
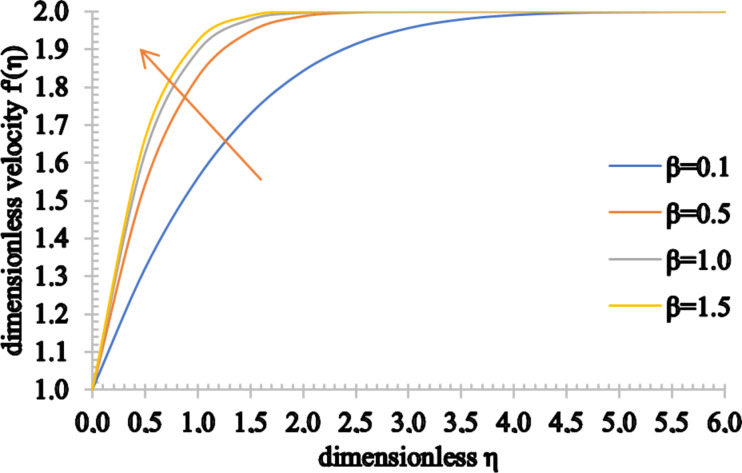
The velocity profile for increasing values of β.

**Figure 9:**
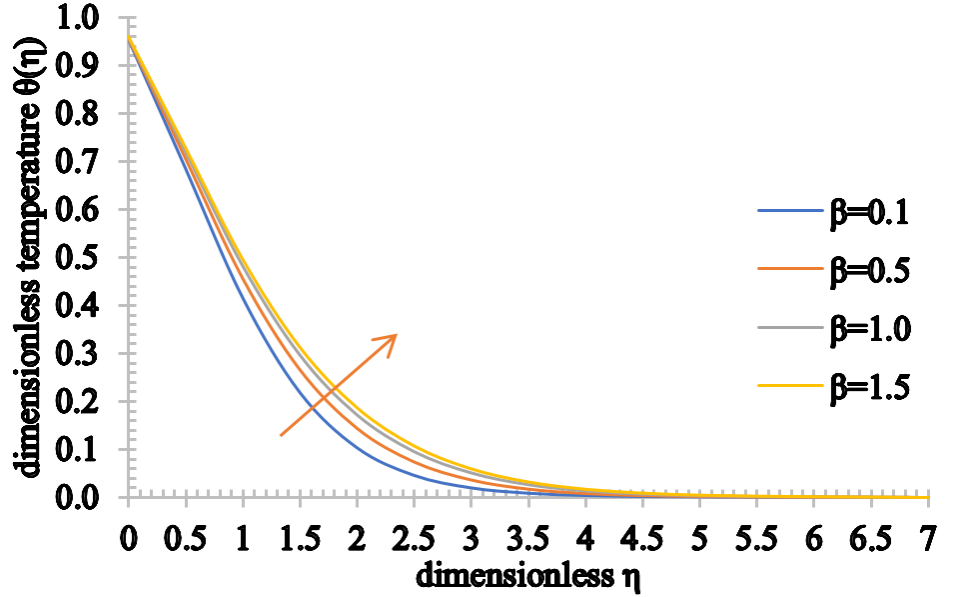
The temperature profile for increasing values of β.

**Figure 10:**
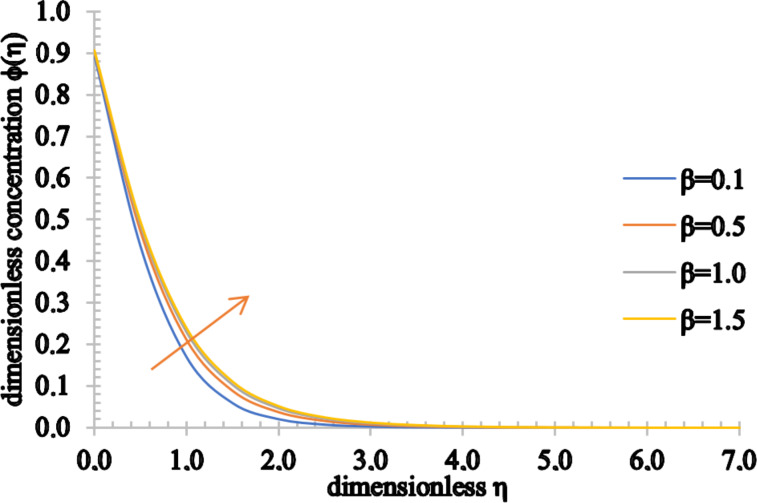
The concentration profile for increasing values of β.

Figures 8–10 show the effect of the Casson parameter on the velocity, temperature and concentration fields. The velocity profile shows an increasing trend when β increases. On the other hand, the velocity boundary layer thickness decreases for higher values of β. This stems from the fact that the plasticity of the Casson fluid increases when β decreases, leading to an increase in the momentum boundary layer thickness. In addition, the values of the temperature distribution as well as the thermal boundary thickness increase when β increases. A rise in the nanoparticle volume fraction and an increase in the concentration boundary layer thickness are observed for higher values of β.

**Figure 11:**
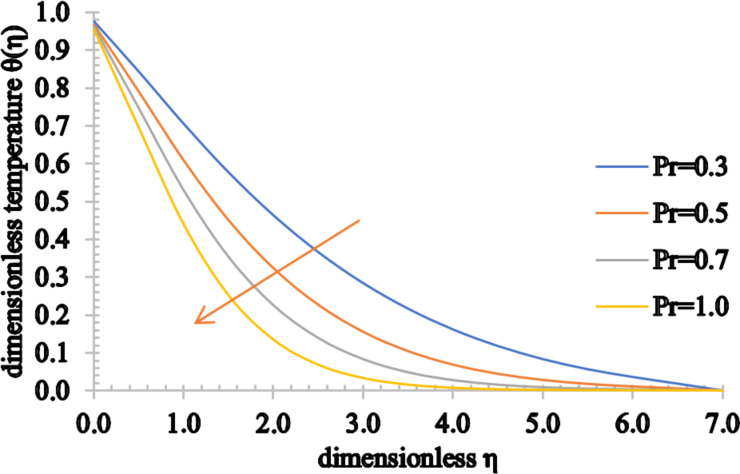
The temperature profile for increasing values of Pr.

**Figure 12:**
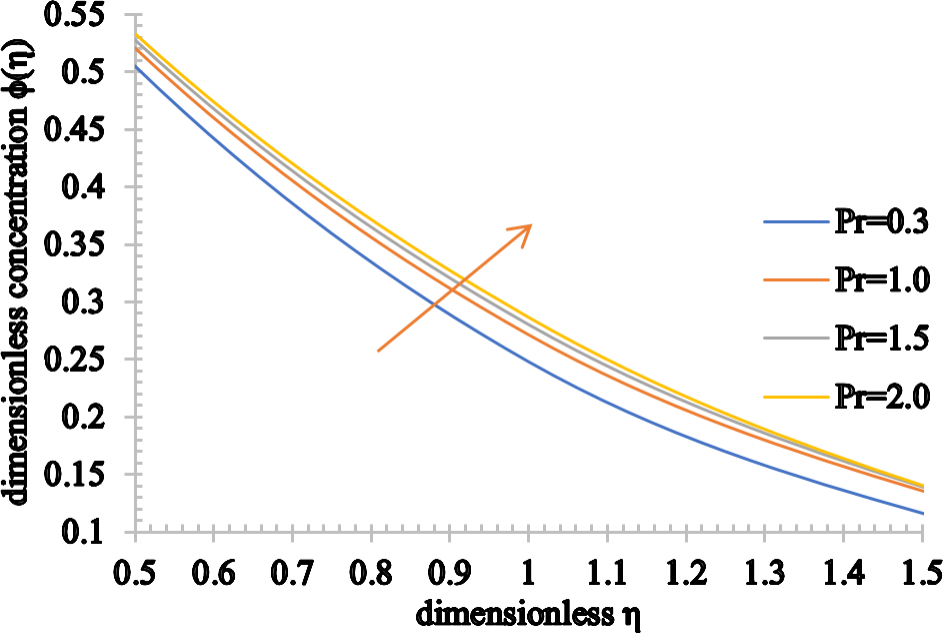
The concentration profile for increasing values of Pr.

Figure 11 and Figure 12 show the effect of Pr on the temperature and concentration distributions. Since Pr is directly proportional to the viscous diffusion rate and inversely proportional to the thermal diffusivity, the thermal diffusion rate is reduced for higher estimated values of Pr. As a consequence, the temperature of the fluid is significantly reduced as well as the thermal boundary layer thickness. Conversely, the nanoparticle volume fraction of the fluid and the concentration boundary layer thickness increase for higher values of Pr.

**Figure 13:**
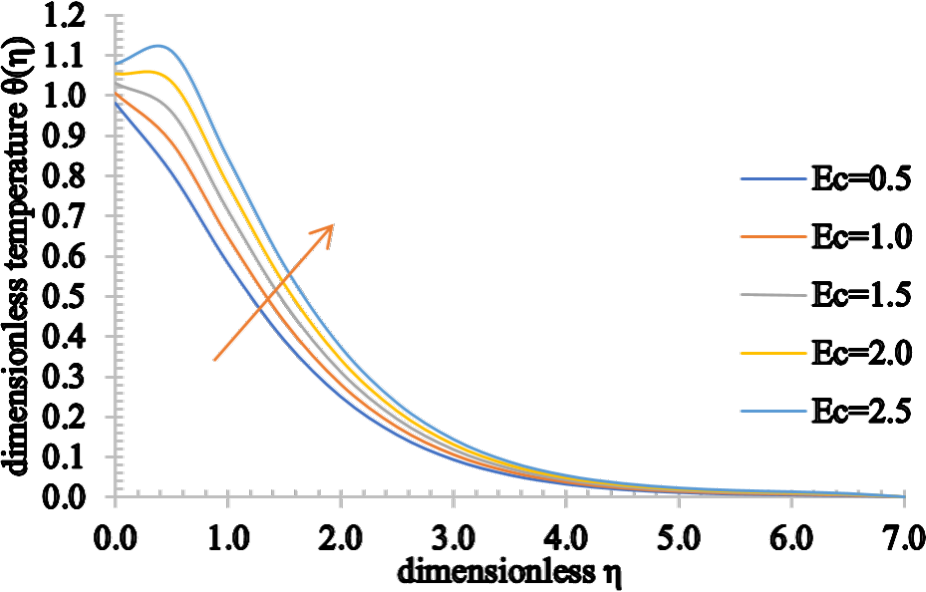
The temperature profile for increasing values of *Ec*.

The outcome of *Ec* on the temperature profiles is characterized in Figure 13. Physically, the Eckert number depicts the relation between the kinetic energy of the fluid particles and the boundary layer enthalpy. The kinetic energy of the fluid particles increases for higher values of *Ec*. Hence, the temperature of the fluid rises marginally and therefore, the associated momentum and thermal boundary layer thickness are enhanced.

**Figure 14:**
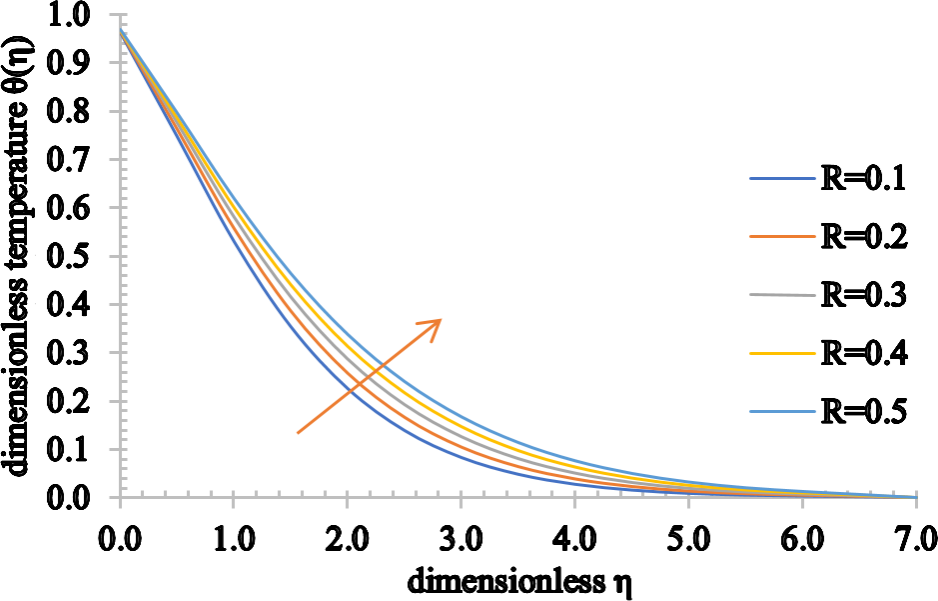
The temperature profile for increasing values of *R*.

**Figure 15:**
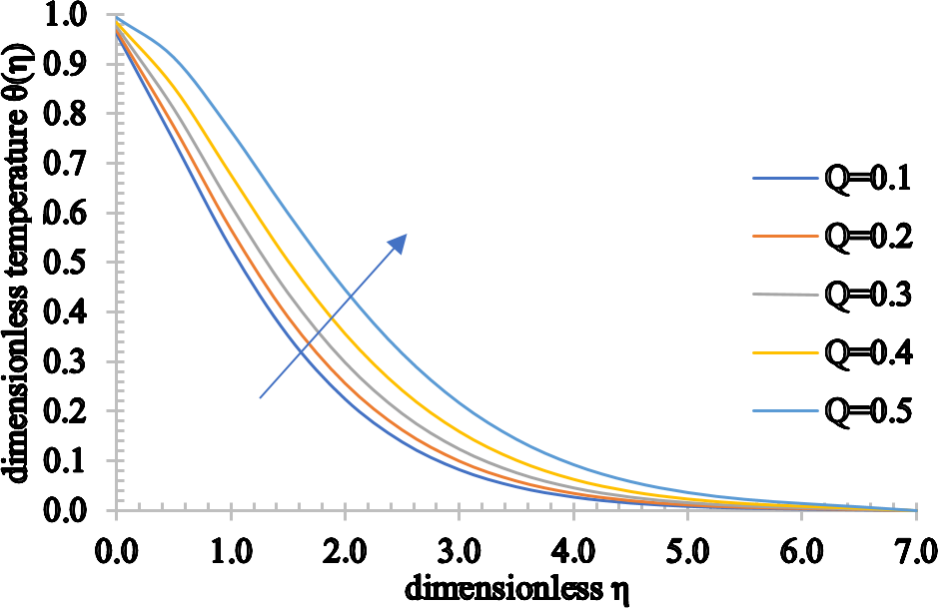
The temperature profile for increasing values of *Q*.

Figure 14 and Figure 15 elucidate the effect of the radiation parameter, *R*, and the heat generation/absorption parameter, *Q*, respectively, on the temperature distributions. Since the heat transfer increases marginally for higher estimated values of *R*, an increment in the temperature of the fluid and also in the thermal boundary layer is seen. However, as the value of *Q* rises, more heat is generated, causing a rise in both the temperature and thermal boundary layer thickness. On the other hand, as the value of *Q* decreases, the absorbed heat results in a decrease of both the temperature and associated thermal boundary layer thickness.

**Figure 16:**
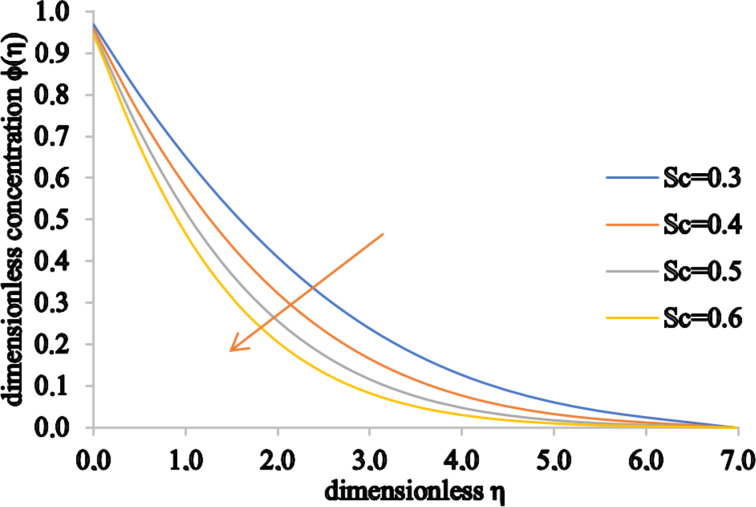
The concentration profile for increasing values of *Sc*.

**Figure 17:**
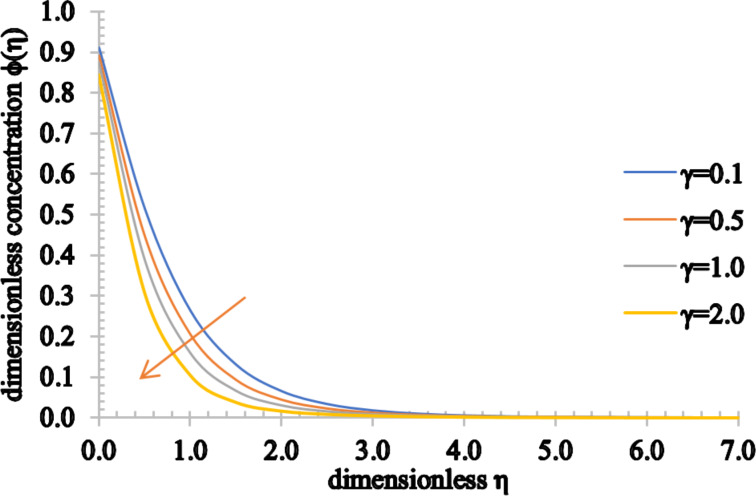
The concentration profile for increasing values of γ.

Figure 16 and Figure 17 show the effects of *Sc* and γ on the concentration fields. The concentration of the fluid decreases for higher values of *Sc*. This behavior stems from the fact that both the Schmidt number and mass diffusion rate have an inverse relation. Therefore, for higher *Sc* values, the mass diffusivity process slows down, decreasing the concentration profile and also the concentration boundary layer thickness. Furthermore, the chemical reaction parameter has a similar effect on the concentration profile. For higher values of γ there is a decrease in the chemical molecular diffusion rate and, consequently, both the concentration of the fluid and the associated concentration boundary layer thickness decrease.

**Figure 18:**
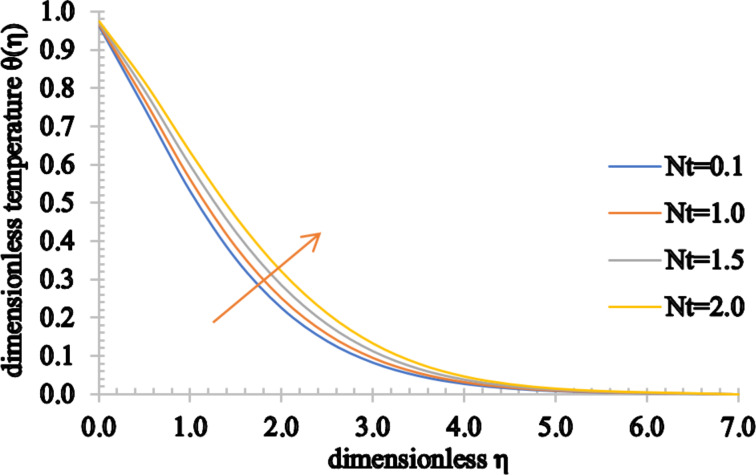
The temperature profile for increasing values of *Nt*.

**Figure 19:**
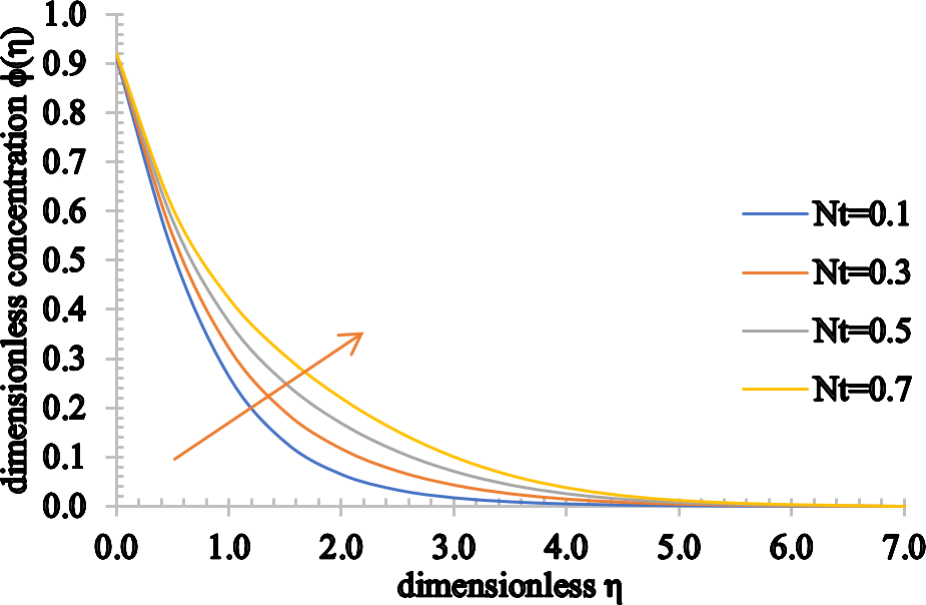
The concentration profile for increasing values of *Nt*.

Figure 18 and Figure 19 show the influence of the thermophoresis parameter on the temperature and concentration distributions. Both the temperature and concentration profiles increase for higher values of *Nt*. In addition to this, an increase in the associated thermal boundary layer thickness and in the concentration boundary layer is noticed.

**Figure 20:**
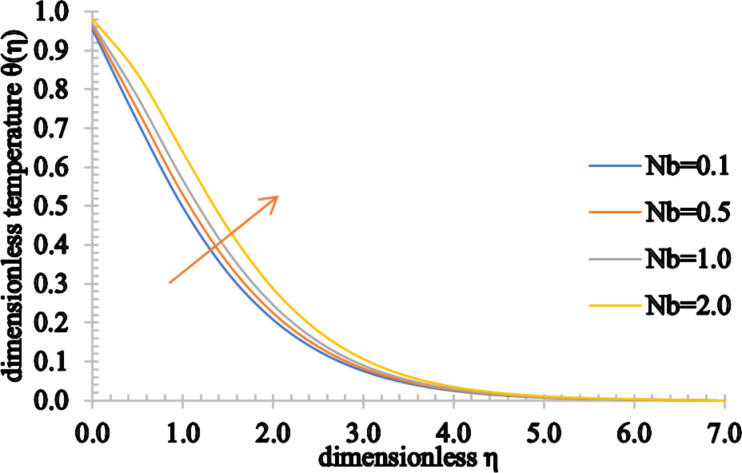
The temperature profile for increasing values of *Nb*.

**Figure 21:**
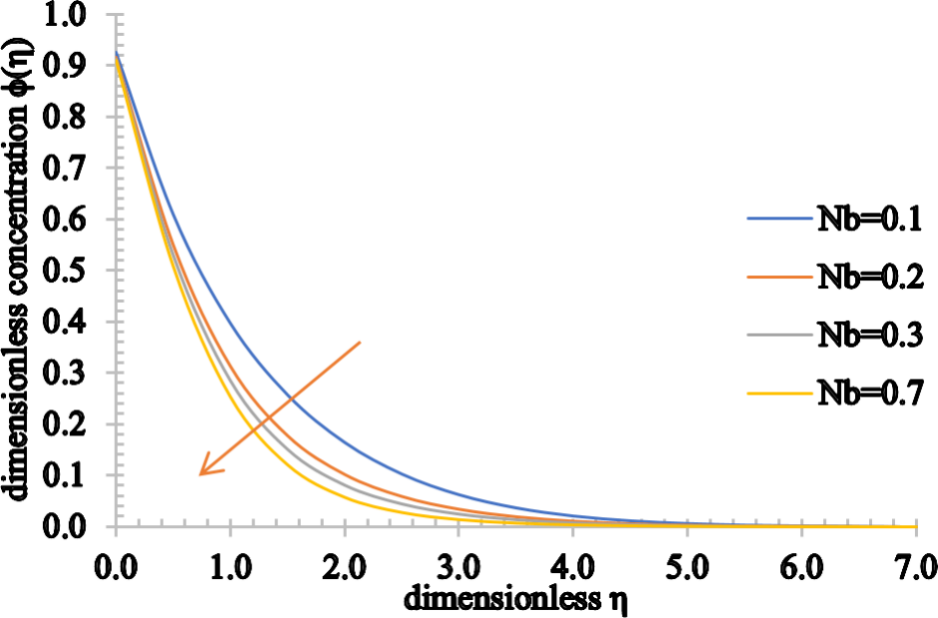
The concentration profile for increasing values of *Nb*.

Figure 20 and Figure 21 display the influence of the Brownian motion parameter on the temperature and concentration distributions. The temperature profile increases marginally for higher values of *Nb*. This happens because, as the value of *Nb* rises, the movement of the nanoparticles increases significantly, increasing the kinetic energy of the nanoparticles. Consequently, the temperature rises and the thermal boundary layer thickness increases. On the other hand, the concentration of the fluid and the concentration boundary layer thickness decrease as *Nb* assumes higher values.

**Figure 22:**
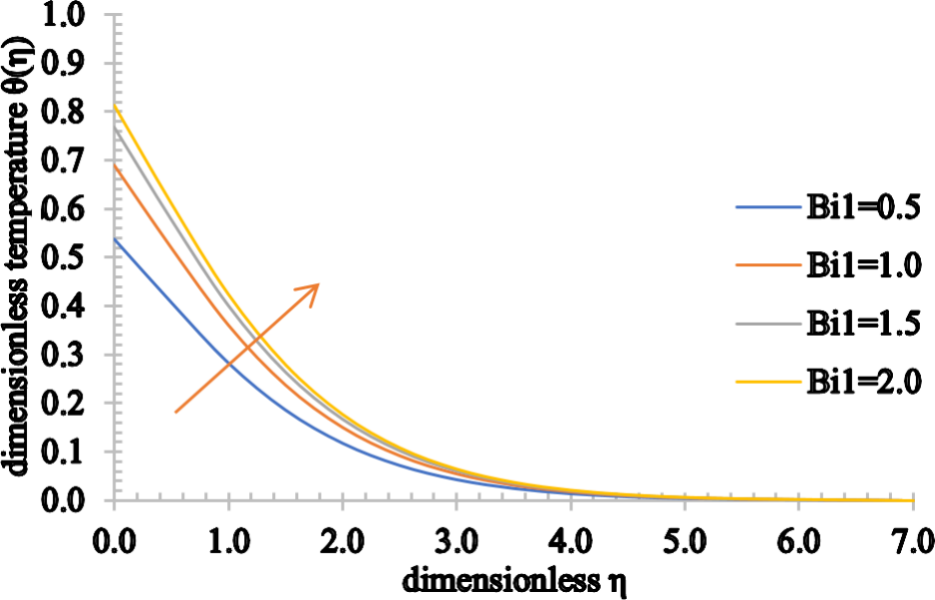
The temperature profile for increasing values of *Bi*1.

**Figure 23:**
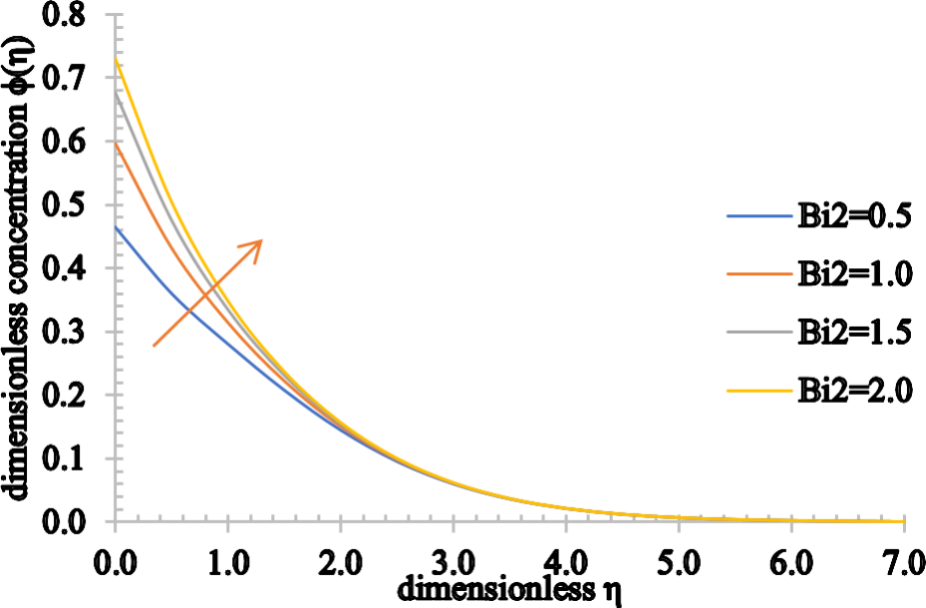
The concentration profile for increasing values of *Bi*2.

The impact of the thermal Biot number on the temperature and concentration distributions and also on the nanoparticle volume fraction is shown in Figure 22 and Figure 23. It is remarkable that the temperature can be observed as an increasing function of *Bi*1 and the concentration of the fluid also increases as *Bi*2 increases. In addition, the associated thermal and concentration boundary layer thickness values are enhanced.

## Conclusion

The numerical investigation of the MHD flow nearby a stagnation point over a radially stretching sheet using Casson nanofluids is presented in this article. Moreover, the radiation effects and the magnetic field are examined. In addition to this, the effects of heat generation/absorption are also explored. It is important to mention that the thermophysical properties vary with the flow rate, temperature and volume concentration. The conversion of nonlinear partial differential equations, describing the proposed flow problem, to a set of ordinary differential equations has been carried out by employing appropriate similarity transformations. The shooting method along with the Adams–Moulton method of fourth order is employed for the numerical treatment. The numerical results show that when the Hartmann number, *Ha*, increases, the velocity decreases whereas an opposite trend is observed for the temperature and concentration fields. In addition, for high values of the Casson parameter, the velocity, temperature and concentration profiles increase. When the Prandtl number increases, the temperature decreases while the concentration of the fluid increases. Additionally, the increase in the Eckert number increases the velocity and the temperature profiles. When the thermophoresis parameter increases, the heat and mass transfer rates also increase. Last but not least, the heat transfer rate also increases with the radiation parameter in Casson fluids.
